# Beyond the curriculum: A gay medical student’s perceptions of health sciences education and healthcare access in KwaZulu-Natal

**DOI:** 10.4102/hsag.v29i0.2656

**Published:** 2024-07-31

**Authors:** Sthembiso P. Mkhize, Sanele Buthelezi, Attah M. Mkhize, Lwandile Tokwe

**Affiliations:** 1 Gauteng City-Region Observatory, University of Johannesburg, Johannesburg, South Africa; 2 Gauteng City-Region Observatory, University of the Witwatersrand, Johannesburg, South Africa; 3 Department of Optometry, Faculty of Health Sciences, University of Johannesburg, Johannesburg, South Africa; 4 Department of Clinical Forensics and Medical Services, Bertha Gxowa Hospital Care Centre, Ekurhuleni, South Africa; 5 Department of Psychiatry and Mental Health, HIV Mental Health Research Unit, University of Cape Town, Cape Town, South Africa

**Keywords:** LGBTIQA+, inclusion, health sciences education, heteronormativity, healthcare services

## Abstract

**Background:**

The prevailing social constructs of cis-heteronormativity and endosexism have structurally marginalised sexual and gender minorities, leading to their limited representation and inclusion in mainstream health systems and health sciences education.

**Aim:**

The study aimed to explore a gay medical student’s perceptions of the health sciences curriculum and their experiences in accessing and utilising healthcare services offered both by the university and externally.

**Setting:**

At a university in KwaZulu-Natal, South Africa.

**Methods:**

This study originates from a larger qualitative study conducted in 2018, involving 12 Lesbian, Gay, Bisexual, Transgender (LGBT)-identifying participants who were selected using purposive and snowball sampling techniques. The larger study used a case study approach to explore how healthcare services meet the sexual health needs of LGBT youth. Among the 12 sampled participants, one participant self-identified as ‘gay’ and was pursuing a Bachelor of Medicine and Bachelor of Surgery. This participant was specifically selected for analysis in this study because of their knowledge of health sciences education and pursuit of a career in healthcare.

**Results:**

Three themes were identified in the participant’s interview, including: (1) navigating healthcare services as a young gay man, (2) silenced voices: the impact of the limited Lesbian, Gay, Bisexual, Transgender, Intersex, Queer, Asexual and + (LGBTIQA+) health education, and (3) challenging the silenced voices.

**Conclusion:**

There is a need for a well-planned curriculum that includes LGBTIQA+ issues to equip healthcare professionals with the knowledge to provide high-quality care to all patients, regardless of their sex, gender, or sexuality.

**Contribution:**

The study provides solid proposals for developing an inclusive healthcare curriculum that considers identities beyond binary going forward.

## Introduction

### Background

The Diagnostic and Statistical Manual of Mental Disorders^1^ previously classified sexual and gender identities beyond binary as mental disorders requiring medical interventions and treatment such as conversion therapies (De Vries, Kathard & Müller 2020; Njenga 2007). Classifications related to intersex variations remain pathologised and treated as medical disorders rather than recognised as natural variations of human biology (Carpenter 2018a). This pathologisation and historical classifications of homosexuality persists even in the development of health sciences curricula. Furthermore, there is limited research on the inclusion of individuals who identify as Lesbian, Gay, Bisexual, Transgender, Intersex, Queer, Asexual, and broadly fluid identities (LGBTIQA+^2^) in health science education as a result of their structural exclusion from medical research and health systems.

Recent evidence suggests that health sciences education continues to use a biomedical approach to teaching sex, sexual orientation, and gender identity (Müller 2013). Consequently, it becomes challenging to situate non-normative identities in their social contexts because there is very little opportunity to question their constructs through social dynamics (De Vries et al. 2020; Müller 2013). For instance, sexuality has seldom been a prominent subject in analysing issues related to access and use of healthcare services, health systems management, or epidemiological research (Müller 2013). Instead, it is primarily taught in the context of reproduction and associated with risks or dysfunctions and not as a predictor of health (Müller & Crawford-Browne, 2013). Similarly, with sex, society often operates under the assumption that there are only two sexes (i.e. male and female), which overlooks the existence of intersex variation (Carpenter 2018b).

Prevailing social constructs such as heteronormativity, cisnormativity, and endosexism have structurally violated sexual and gender minorities by perpetuating limited representation within the mainstream narratives of health systems and health sciences education (Bleasdale et al. 2024; De Vries et al. 2020). This exclusionary pattern has had far-reaching consequences, impacting both the quality of health sciences education provided to future healthcare professionals, the care that healthcare professionals provide to LGBTIQA+ patients, and the well-being of LGBTIQA+ individuals. One of the primary ways in which these normative social constructs manifest in health sciences education is through the underrepresentation of LGBTIQA+ experiences and perspectives in health sciences curricula, textbooks, and lecture discussions (Bleasdale et al. 2024; Gordon & Mitchell 2019). This lack of representation not only fails to adequately prepare future healthcare professionals to provide sensitive and effective care to LGBTIQA+ patients but also sends a message that these individuals and their health issues are less important.

Identifying as ‘LGBTIQA+’ has automatically placed many queer people at a heightened risk of experiencing numerous adverse health outcomes as a result of their vulnerabilities, discrimination, and structural violence (Medina-Martínez et al. 2021). Earlier work by Silvers (2004) asserts that individuals who belong to a specific group with distinct barriers to accessing care across various circumstances are less equipped than others to protect their own needs and are at a relatively elevated risk of experiencing adverse psychological, physical, and social health outcomes. Ekmekci (2017) classifies queer people as a vulnerable population within bioethical discussions because of their potential susceptibility to exploitation in clinical research studies. This exploitation primarily stems from the challenges they experience as a result of social exclusion, limited social support, low self-esteem, elevated rates of physical and mental health disorders, and inadequate access to appropriate healthcare services. The increased vulnerability within queer people is then exacerbated by the pervasive invisibility of their experiences throughout various facets of society, from societal dynamics to health sciences education to the healthcare system.

Evidence suggests that discrimination, stigmatisation, and a lack of understanding of the health experiences of LGBTIQA+ patients within healthcare settings can inhibit these individuals from seeking necessary care or being open about their health concerns (Cele, Sibiya & Sokhela 2015; Mkhize & Maharaj 2021). In addition, queer people remain at a higher risk compared to their heterosexual counterparts of experiencing poor health outcomes such as contracting sexually transmitted infections (STIs) including human immunodeficiency virus (HIV), and experiencing elevated depressive disorders (Hafeez et al. 2017; Jankovic et al. 2020; Müller 2017; Wood, Salas-Humara & Dowshen 2016). In South Africa, where the constitution is progressive and protects the rights of queer people, recent studies reveal persistent discrimination against them within the healthcare system (Mkhize & Maharaj 2023; Müller & Daskilewicz 2019). This discrimination further widens the disparity in health outcomes, an increased burden of diseases, and difficulty in finding culturally competent healthcare services, which are all driven by normative and exclusionary practices from those in positions of power (Daly 2022; Noonan et al. 2018).

Globally, the gaps in health sciences curricula have been documented. A study conducted by Müller et al. (2017) in Malawi and South Africa revealed limited inclusion of LGBTIQA+ health-related topics in the medical and nursing curricula, as there was no support from the institution mandating the inclusion of such topics. Similarly, Müller’s (2013) study conducted at the University of Cape Town’s medical school found no dedicated module for teaching Lesbian, Gay, Bisexual and Transgender (LGBT) health. In one study conducted at a medical school in the United Kingdom (UK), 85% of the participants indicated a lack of LGBTIQA+ education in the curriculum, and a majority also indicated that they would not make it a routine to ask about sexuality or gender needs during consultations because they lacked confidence regarding their knowledge of LGBTIQA+ health needs and the use of appropriate terminology (Parameshwaran et al. 2017). In another study conducted in the UK, 29% of transgender participants reported that their gender identity was not acknowledged or respected in mental health settings but instead treated as a symptom of a mental illness (Ellis, Bailey & McNeil 2015). Furthermore, Ellis et al. (2015) revealed that gender-diverse individuals felt discomfort because of being misgendered, deadnamed, and mispronounced in most communications within the mental health facility. This parallels an earlier study in which gender-diverse individuals experienced limited access to holistic healthcare because of a lack of awareness about transgender health issues (Newman-Valentine & Duma 2014). In contrast, an earlier study conducted by Kelley et al. (2008) highlighted the fact that medical students who received teaching and learning experiences on LGBTIQA+ health-related topics felt more comfortable and well-equipped to provide care to LGBTIQA+ patients because they understood the relevance of cultural sensitivity and its impact on patient outcomes. Based on the aforesaid arguments, healthcare professionals could benefit from understanding some of the issues shared by a medical student who identifies as part of the LGBTIQA+ community.

### Aim

The study aims to explore a gay medical student’s perceptions of the health sciences curriculum and their experiences in accessing and utilising healthcare services offered both by the university and externally.

## Research methods and design

### Context, design and sample size

This study stems from a larger qualitative study conducted in 2018 (Mkhize 2019), which used a case study approach to explore how healthcare services meet the sexual health needs of LGBT youth in KwaZulu-Natal, South Africa. The larger study conducted in-depth interviews with 12 registered students from the University of KwaZulu-Natal (UKZN), who were between 18 and 24 years old, self-identifying as either lesbian, gay, bisexual, or transgender, and selected using purposive and snowball sampling techniques. However, one student identifying as ‘gay’ and studying towards a Bachelor of Medicine and Bachelor of Surgery was purposefully selected for analysis of this study because of the student’s knowledge related to health sciences education and pursuit of a career in healthcare. A case study approach facilitated an in-depth interviewing process and analysis of one participant’s experiences, which has been recommended for validity in prior literature (Quintão et al. 2020). The chosen medical student was assigned the pseudonym ‘Muzi’ to facilitate the presentation of findings. During fieldwork, Muzi was 24 years old and in his final year and possessed sufficient knowledge to reflect on his perceptions of the health sciences curriculum and his experiences in accessing and utilising healthcare services.

### Ethical considerations

Ethical approval for the larger qualitative study was obtained from the UKZN Humanities and Social Sciences Research Ethics Committee (HSS/0302/018M). The study was mindful of not disclosing the sexual or gender identities of participants who were not yet comfortable with public disclosure, and all participants signed an informed consent form to confirm voluntary participation. Unique identifiers and pseudonyms were used in place of their real names to ensure anonymity. Considering the potential negative impact on participants’ mental health, post-interview psychological support arrangements were made with the university’s student counselling unit.

### Analysis

A thematic analysis based on an interpretive approach was used for data gathering and analysis to explore Muzi’s perceptions of the health sciences curriculum and their experiences in accessing and utilising healthcare services offered by the university and externally. The qualitative data collected underwent three significant phases of coding. The first phase involved transcribing the data verbatim (in the language in which they were conducted) and translating parts of interviews transcribed from a local language (IsiZulu) into English to facilitate coding and ensure comprehensibility for reporting purposes. The second phase encompassed coding line by line to identify themes and patterns within the study, while the third phase entailed categorising the codes into salient themes. The findings presented in the Results section are based on a narrative account of Muzi’s interview.

## Results

Three themes were identified in Muzi’s interview, including: (1) navigating healthcare services as a young gay man, (2) silenced voices: the impact of the limited LGBTQIA+ health education, and (3) challenging the silenced voices. These themes shed light on topics such as cultural competence, sensitivity to diverse sexual orientations and gender identities, and integration of relevant healthcare issues faced by the LGBTIQA+ community within health sciences education.

### Navigating healthcare services as a young gay man

Muzi initiated his engagement with sexual healthcare services upon becoming sexually active at the age of 19 years, which was the same period that coincided with his enrolment at UKZN’s medical school. Muzi’s primary point of accessing healthcare services was the university campus clinic, which he regarded as highly sufficient to meet his sexual healthcare needs compared to clinics outside the university:

‘I use the campus clinic, most of the time. I never really need or use the [*public*] hospital for my sexual health care needs. The clinic is just sufficient, and that is where I get my condoms, lubes [*lubricants*] and … even when I have concerns like when I see a pimple in my genitals, that is where I usually go.’ (Muzi, medical student, 24 years old)

Muzi reflected on how his experience with healthcare professionals at the university campus clinic and outside the university evolved. Initially, he reported feeling uneasy discussing his sexual health concerns with an older female nurse from the university clinic. This discomfort stemmed from the fear of being judged because of his sexuality and the notion of talking about sexual matters with an older woman who could be a parent, especially since he was still in the closet^3^. However, as years progressed, 24-year-old Muzi found a gradual change because the more he accessed the health services, the more he became comfortable with the service provided. He also noticed that generally, healthcare professionals are taught about the significance of confidentiality, and he appreciated the respect that the specific older female nurse consistently showed him each time he sought care at the campus clinic:

‘My experience [*at the campus clinic*] was not that bad. So, we have this female nurse, she is older and now I feel like I am always very comfortable speaking to her about my sexual health concerns. The only time I felt uncomfortable was the time I was exposed to HIV. Since I was bottoming [*receptive partner*], it felt so difficult and uncomfortable opening up to the nurse about the incident because I was still in the closet. I just thought she would judge me, considering the fact that she was older and female. It also felt like I would be speaking to my mother, telling her about my sexual problems, which is a taboo in our African culture.’ (Muzi, medical student, 24 years old)

### Silenced voices: the impact of the limited LGBTQIA+ health education

While highlighting the positive aspects of his experiences with healthcare services, Muzi also shed light on his challenges when utilising services outside the university. Muzi emphasised the limited responsiveness of external healthcare services in addressing his needs as a gay patient. He mentioned that many healthcare professionals outside the university lacked awareness, sensitivity, and training about the unique needs of LGBTIQA+ patients, as a whole, and the skills on how to provide appropriate care for them. Furthermore, he observed that the environment of the external healthcare facilities often appeared to cater primarily to heterosexual and cisgender patients, with little to no representation, inclusion, or diversity in promotional materials that would signal friendliness towards LGBTIQA+ patients:

‘There are limited resources for sexual health measures for LGBT patients, and most of the [*health*] staff are just not aware of what we need as the LGBT community.’ (Muzi, medical student, 24 years old)

Muzi also reflected on his negative encounter with a healthcare professional who was deeply religious from the external healthcare facility, noting that he was unable to offer him adequate care because of their religious beliefs on homosexuality and same-sex behaviours such as the choice of physical appearance:

‘I was denied care by a very religious male nurse because of the way I dressed.’ (Muzi, medical student, 24 years old)

In addition, he also highlighted the challenge of educating medical students who hold strong religious beliefs and may be resistant to learning about LGBTIQA+ health topics that conflict with their religious teachings:

‘It is very hard to teach and change someone about their religion, especially when they grew up in that religion. This is something we can never change, but I think we should emphasise to religious people that they should not let their religious beliefs ruin their careers and the service they should be providing to other people because of their sexual preferences or gender differences.’ (Muzi, medical student, 24 years old)

Muzi also linked his negative encounter with healthcare services with the limited inclusion of LGBTIQA+ health-related topics in health sciences education. During the interviews, Muzi mentioned that UKZN’s medical school covers certain aspects of diversity and inclusion but does not delve deeply into the real-world health experiences of LGBTIQA+ patients:

‘They do not teach these things [*LGBT-related sexual health topics*] in Medical School. If they do, they just do not dwell much on the practical experiences [*realities*] of LGBT people. You can even go to medical school now and ask students if they know of any health complications specific to LGBT people, they will tell you that they have no clue.’ (Muzi, medical student, 24 years old)

Muzi expressed that the lack of healthcare professionals who offer appropriate care can be attributed to the lack of skills and experience of some lecturers and healthcare professionals who were not exposed to LGBTIQA+ health topics in their curriculum during their years of study:

‘We still lack lecturers and medical professionals who would teach us these things, the lecturers who are hired are just teaching from a wide perspective and are not focusing on the specifics of how to provide care for someone identifying as LGBTIQA+.’ (Muzi, medical student, 24 years old)

### Challenging the silenced voices

Muzi shared his perspective on how to address the exclusion of LGBTIQA+ health-related topics in health sciences education. He emphasised that teaching and education remain powerful tools for challenging the cis-heteronormative systems of healthcare and higher education. One of Muzi’s suggestions is to introduce a mandatory module that covers various health aspects disaggregated by characteristics of sex, gender, and sexuality across all health science faculties. In his view, this approach would enable medical students to gain a deeper understanding of societal diversity and to promote cultural competence through intersectional lenses:

‘Teaching and educating the mind is always the greatest weapon … I think there should be a compulsory type of thing, maybe a module or a seminar that should be attended by the university medical staff and lecturers as well as students.’ (Muzi, medical student, 24 years old)

Muzi also emphasised that South Africa’s constitution has not fully guaranteed equality for the LGBTIQA+ community who access and use healthcare facilities. He strongly stressed the importance of LGBTIQA+ patients being aware of their rights to report instances of mistreatment and discrimination that they may encounter within healthcare facilities:

‘LGBTIQA+ people should also be aware of their rights and the legislation that was established by the constitution, they should be able to make use of their rights and report discriminating health providers so that new staff would be hired, a staff that would know that all human lives matter.’ (Muzi, medical student, 24 years old)

As mentioned earlier, most health services are based on normative principles and primarily cater to the health needs of heterosexual patients. To address this issue, Muzi recommended that the health systems invest in creating resources that promote inclusion. Muzi believed this could encompass promotional materials such as posters and pamphlets to educate healthcare professionals about the LGBTIQA+ community and portray the facilities as welcoming and inclusive spaces:

‘I also think there should be billboards, posters and pamphlets in the health centres to show that the Department of Health is also on our side and to assure us that our needs would be met if we access a particular clinic.’ (Muzi, medical student, 24 years old)

## Discussion

From Muzi’s experiences, healthcare professionals at the university clinic were seen as having a better understanding of the needs of LGBTIQA+ students compared to those practising outside the university. One of the reasons behind this perception is that healthcare services provided outside of the university tend to be designed with a cis-heteronormative approach, which primarily addresses the needs of heterosexual and cisgender patients. The existing evidence suggests that healthcare systems worldwide have traditionally been oriented towards serving the heterosexual and cisgender population and are influenced by normative-based promotional resources and a one-sided healthcare environment, which impact how healthcare professionals approach care for LGBTIQA+ patients (Hayman et al. 2013; Meads et al. 2019; Mkhize & Maharaj 2023; Stewart & O’Reilly 2017).

In many traditional African cultures, discussing sexual matters with elders has been regarded as taboo (Hildebrand, Ahumada & Watson 2013; Munyai et al. 2023). In this study, the participant initially indicated hesitance in discussing their sexual concerns with an older healthcare professional because of their belief that such conversations were considered taboo within their cultural norms. This finding aligns with previous research conducted by Alli, Maharaj and Vawda (2013) and Munea et al. (2022). Both studies found that in African cultures, young people may be reluctant to discuss sexual and reproductive health issues with older individuals and healthcare professionals because it can be perceived as disrespectful and contrary to cultural norms.

It has been consistently demonstrated that healthcare professionals often lack the necessary knowledge and training to deliver culturally competent care to LGBTIQA+ patients (Fish & Evans 2016; Matthews & Van Wyk 2018; Smith et al. 2019). In this study, the participant attributed the inadequate provision of appropriate care that they received in health facilities outside the university to the lack of culturally competent and sensitive training on LGBTIQA+ health-related topics for those healthcare professionals. In addition, the participant not only gave an example of their exposure to general topics related to diversity and inclusion in the curriculum provided at medical school but also noticed that there was no depth and comprehensiveness of LGBTIQA+ health-related topics in the curricula. Müller (2013) also found that despite the inclusion of some LGBTIQA+ health-related topics in existing courses within the medical school, there was no formal structure in place to coordinate its integration into the curriculum. This lack of structure diminished its effectiveness in preparing medical students to provide adequate care for LGBTIQA+ patients (Müller 2013).

Some healthcare professionals fail to provide adequate care because of ignorance and personal beliefs (Meads et al. 2019). The participant observed that healthcare professionals lacked the necessary skills to be sensitive to him as a gay patient. He recounted an incident where a healthcare professional from an external health facility made judgements based on his choice to wear nail polish and colourful clothing. Consequently, he was denied care and this denial was rooted in the provider’s religious beliefs. Similarly, Cele et al. (2015) found that sexual and gender minorities faced judgement because of their personality and physical appearance, including the way they dressed, spoke, or carried themselves. In addition, they reported that the religious beliefs of healthcare professionals were a barrier to receiving appropriate care. South Africa stands out as one of the world’s most culturally and religiously diverse countries and some healthcare professionals express their strong beliefs openly. The 2016 Health Professionals Council of South Africa (HPCSA) guidelines for good practice in the healthcare professions may have ‘*carelessly*’ empowered healthcare professionals who were inclined to impose their personal beliefs, as there was no mention of how healthcare professionals should treat and respect patients regardless of their sexual orientation (HPCSA 2016). This suggests that inadequate training and education were more likely provided to healthcare professionals before the time of the participant’s interview in 2018, which allowed healthcare professionals the discretion to withhold and deny care from individuals whose beliefs conflicted with their own. However, it is worth mentioning that the revised 2021 document indicates a shift in approach, as it emphasises that the maintenance of good professional practice should be rooted in core ethical values, including respect and tolerance for patients with distinct beliefs. Furthermore, healthcare professionals are explicitly advised to ensure that their personal beliefs do not prejudice the healthcare provided to patients (HPCSA 2021).

### Recommendations: Fostering inclusivity in health sciences education

There is certainly no doubt that promoting inclusivity of LGBTIQA+ health-related topics in health sciences education fosters culturally competent healthcare, enhances access to care, improves patient–provider communication, and reduces health disparities experienced by LGBTIQA+ patients (Gitlin, Demla & Sewell 2021; Guest & Weinstein 2020; Müller 2013; Sanchez et al. 2017). Higher education institutions play an essential role in supporting curricula development that can reach all groups, including individuals who identify as LGBTIQA+. The 2017 report by UKZN on the Quality Enhancement Project Phase II: Curriculum marked the initial step for the university in showcasing its commitment to address critical issues concerning its curriculum (UKZN Teaching and Learning Office 2017). While this report acknowledges that higher education curricula have historically been designed and implemented in a cis-heteronormative approach, it emphasises the significance of undertaking comprehensive curriculum reforms that accommodate the unique needs of queer people. However, disparities persist within the UKZN’s College of Health Sciences, where the curriculum remains predominantly theoretical and is administered in field-specific manners (UKZN Teaching and Learning Office 2017). This places an emphasis on the urgency of infusing a sense of cultural humility into health sciences education to purposefully transform the educational structure for a better understanding of the healthcare needs of LGBTIQA+ patients. In addition, this transformation involves amplifying the voices and experiences of queer people within the curriculum.

In South Africa, higher education institutions are known for their emphasis on promoting equality, diversity, and inclusion in the realm of teaching and learning (Dalton, Mckenzie & Kahonde 2012; Matthyse 2017). The 1997 White Paper on Higher Education highlighted sexual orientation as one of the focal points for creating a safe and secure campus environment with an aim to discourage harassment and hostile behaviour directed at queer people (Hames 2007). This is relevant to the participant’s experience of feeling safer and more welcomed at the campus clinic compared to the external healthcare facilities. This same principle should extend to the curriculum offered to health science students. Health sciences education curricula must foster reflective practice on the cis-heteronormative discourse, as this can allow both health educators and students to examine their own biases and judgements critically and understand the origin of these biases so they strive towards creating safer and inclusive spaces for queer people (Harbin, Beagan & Goldberg 2012; Ross & Setchell 2019). One way to achieve this is by introducing a mandatory module (also suggested by the participant) in health sciences education that encompasses an understanding of concepts covered by Killermann (2017) in the ‘Genderbread Person’, appreciating complex dimensions of a patient’s identity through intersectional lenses by Crenshaw (2013), and exposing health science students to necessary literature and practical experiences to enhance their understanding of social determinants of LGBTIQA+ people related to specific disease risks and access to care. In addition, incorporating inclusive case studies that feature LGBTIQA+ people in the curriculum can help medical students apply their knowledge to real-world scenarios and learn to provide inclusive and sensitive care.

During the interview, the participant reported experiencing the external health facilities as highly exclusive and non-accommodating to LGBTIQA+ patients. Therefore, it is imperative that health environments, such as hospitals and clinics where health science students undergo practical training and internships, expose them to spaces that are inclusive and LGBTIQA+ friendly. To ensure this, one effective approach is the implementation of inclusive data collection forms that encompass a broader spectrum of sex beyond the binary to accommodate intersex patients, sexual orientations, and gender identities within healthcare facilities. Baker (2012) emphasises the significance of integrating such forms into healthcare systems to show its role in maintaining a comprehensive health history for all patients and to help healthcare professionals determine the specific health needs of LGBTIQA+ patients. Furthermore, Zeeman et al. (2019) also share an additional strategy for ensuring that services are tailored to include the needs of LGBTIQA+ patients. Just as free cervical cancer screening for women is advertised and promoted in healthcare facilities, Zeeman et al. (2019) emphasise the importance of openly promoting and providing anal cancer screenings for gay and bisexual men because they are twice as likely to be diagnosed with anal cancer. In many ways, this would reinforce the necessity for the South African healthcare system to cultivate an inclusive environment by displaying promotional brochures and educational materials addressing LGBTIQA+ health concerns in public health facilities.

Müller (2013) asserts that recent policies in South Africa have acknowledged the need to incorporate LGBTIQA+ education into health sciences curricula. However, there is a lack of understanding regarding how to integrate LGBTIQA+ health-related topics effectively into health sciences education. To address this, it would be advisable to promote the development of national standards and coherent educational initiatives for medical and health sciences faculties in South Africa. This will ensure the establishment of an integrated teaching and learning framework that can be nationally adopted across all health sciences education programmes. While guidelines for healthcare professionals are already provided by HPCSA, it is essential to establish national standards for higher education departments of health sciences. The implemented national standard should guide the curriculum design that compels all lecturers and educators to adhere to teaching about the experiences of LGBTIQA+ in specific modules to ensure that the curriculum is not biased towards the patients’ experiences. In addition, this national standard can rely on the voices of minority groups and various stakeholders while drawing on queer theory and intersectional feminism to develop LGBTIQA+-friendly curricula to address the various ways in which LGBTIQA+ patients encounter discrimination. In this way, the health sciences education can be queer(ed) to embody the practice of deconstruction by adopting non-binary teaching methods to guarantee a curriculum that does not only favour normative behaviours of sex, sexuality, and gender.

### Strengths and limitations

This study adopted a case study approach to capture the perceptions and experiences of one participant. Some may view this as a limitation, as conclusions drawn from a single voice may not adequately represent the diversity of perceptions and experiences within a larger group. However, the participant’s narrative is unique, as they were both a medical student studying to become a doctor and a gay individual who accessed and utilised healthcare services for their sexual needs during their medical training. Future research could replicate a similar study and gather qualitative data from a larger sample of medical students across all universities in South Africa to gain a deeper understanding of their perspectives and to observe changes made in the health sciences curriculum as this study collected data in 2018.

The findings may appear biased towards the ‘G’ in the LGBTIQA+ acronym because the participant is gay and they reflected on their own experiences. However, the rationale for grouping the entire community with fluid and diverse identities (i.e. LGBTIQA+) originated from the shared challenges they face as a community, including being denied rights and experiencing poorer health outcomes compared to their heterosexual and cisgender counterparts. Hence, the findings and recommendations are generalisable to the entire LGBTIQA+ community because they encounter similar challenges as a minority group.

This study contributes to the limited research on the inclusion of LGBTIQA+ health-related topics in the South African context. This gap has led to a healthcare science curriculum primarily designed with approaches to cis-heteronormativity. Without sufficient evidence, necessary interventions related to developing and strengthening cultural competence programmes cannot be implemented in higher education and healthcare systems. Hence, the findings and recommendations provided in this study are crucial contributions to addressing these gaps in research, as they offer actionable steps towards integrating LGBTIQA+ health topics into the curriculum and fostering inclusivity within healthcare education and practice in South Africa.

## Conclusion

This study draws on the perceptions of a medical student identifying as ‘gay’ regarding the health sciences curriculum and their experiences in accessing and utilising healthcare services offered by the university and externally. The participant’s narratives suggest that there are still gaps in the provision of LGBTIQA+ health-related topics in health sciences education. This study also provided several recommendations, including the implementation of inclusive teaching practices to enhance learning and foster the development of the necessary knowledge, attitudes, and skills required for providing quality care to LGBTIQA+ people. In addition, developing a specific national standard for higher education departments of health sciences can guide curriculum design that is not biased. This study places a crucial emphasis on producing healthcare professionals who possess awareness, sensitivity, and knowledge in providing care to LGBTIQA+ patients to reduce the health disparities currently faced by this community.

## Footnotes

1 A globally influential reference for diagnosing mental disorders. Access here: https://www.psychiatry.org/psychiatrists/practice/dsm.

2 The acronym ‘LGBTIQA+’ is used to explicitly include all individuals with non-normative sex, sexual, and gender identities. However, on occasion, the term ‘queer’ will be used to acknowledge the infinite number of complexities and fluid identities that exist beyond the limited binary sex/gender/heterosexual categories deemed legitimate by society.

3 The term ‘closet’ in the context of LGBTIQA+ individuals refer to the state of not openly acknowledging or disclosing one’s sexual orientation because of fear of rejection and discrimination.
